# An Unusual Occurrence of Hepatic Granulomas and Secondary Sitosterolemia in Turner Syndrome

**DOI:** 10.1155/2015/186718

**Published:** 2015-01-29

**Authors:** JayaKrishna Chintanaboina, Pragnesh R. Shah, Thomas R. Riley

**Affiliations:** ^1^Penn State Hershey Medical Center, Penn State College of Medicine, Division of General Internal Medicine, 500 University Drive, Hershey, PA 17033, USA; ^2^Memorial Hermann Sugar Land Hospital, 17500 West Grand Parkway, Sugar Land, TX 77479, USA; ^3^Penn State Hershey Medical Center, Penn State College of Medicine, Division of Gastroenterology and Hepatology, 500 University Drive, Hershey, PA 17033, USA

## Abstract

Although abnormal liver function tests occur in 50–80% of cases with Turner syndrome, there are no previous reports of overt hepatic disease or hepatic granulomas associated with Turner's syndrome. We report three cases of Turner syndrome associated with hepatic granulomas with a wide range of liver dysfunction. Of the three patients, first patient underwent liver transplantation; second patient remained stable on immunosuppressants; and third patient died from complications of decompensated liver cirrhosis as she declined liver transplantation due to multiple comorbidities. One patient had sitosterolemia, a rare inherited autosomal recessive disorder of cholesterol metabolism, after she ingested *β*-sitosterol supplement and had worsening liver function tests and lipid panel. She had remarkably abnormal lipid panel that responded to ezetimibe and by stopping the *β*-sitosterol supplement.

## 1. Introduction

Turner syndrome is one of the more common genetic disorders, associated with abnormalities of the X chromosome and occurring in about 50 per 100,000 live female births. Turner syndrome is usually associated with reduced height and gonadal dysgenesis and thus insufficient circulating levels of sex hormones and infertility. Turner syndrome is often associated with certain endocrine and metabolic disturbances such as glucose intolerance, diabetes, thyroiditis, hypertension, and elevated liver enzymes. Elevated liver enzymes are seen in 50–80% of patients with Turner syndrome; however, no overt hepatic disease has been identified [1, 2]. Hepatic granulomas are found in a variety of disorders. They may also be an incidental finding on otherwise normal liver biopsy. The most important causes of hepatic granulomas are systemic infections, malignancy, drugs, autoimmune disorders including sarcoidosis, and idiopathic. The clinical consequences of the hepatic granulomas are variable and range from asymptomatic incidental finding to abnormal liver function tests (LFTs) with cholestatic liver pattern, hepatic vein thrombosis, and cirrhosis [3–5]. Association of Turner syndrome with sarcoidosis and granulomatous process has been described only once previously by Tsuji et al. [6]. There are no previous reports of the association of Turner's syndrome with hepatic granulomas. Here we describe three cases of Turner syndrome associated with hepatic granulomas with one of the patients having Sitosterolemia, a genetic disorder of cholesterol metabolism.

## 2. Case Series

### 2.1. Case  1

A 44-year-old Caucasian female with past medical history of Turner syndrome, hypothyroidism, and pulmonary sarcoidosis that was diagnosed by a computed tomography scan of the chest and bronchoscopy with biopsy about 15 years prior had persistent abnormal LFTs for several years. She remained asymptomatic from sarcoidosis and thus never received therapy. Four years after diagnosis of pulmonary sarcoidosis, she was found to have abnormal LFTs and was referred to our hepatology clinic. Physical examination showed a short statured female without any jaundice, asterixis, or any stigmata of end-stage liver disease. Liver was nontender and measured 8 cm in the midclavicular line. Spleen was not palpable. Laboratory data is shown in Table 1. Other labs including anti-mitochondrial antibody (AMA), anti-smooth muscle antibody (ASMA), and ferritin levels were negative. A percutaneous liver biopsy showed noncaseating granulomas with multinucleated giant cells without any evidence of anaplasia; stains for iron, fungus, and acid-fast bacilli were negative.

LFTs were followed periodically and remained stable but mildly elevated consistent with cholestatic liver disease pattern. At one point, she developed jaundice without any pruritus or other constitutional symptoms and was noted to have markedly worsening LFTs and lipid profile after she took *β*-sitosterol supplements for several months. Repeat work up for abnormal LFTs including hepatitis panel and autoimmune panel was unremarkable. She was diagnosed with sitosterolemia, an autosomal recessive disorder associated with increased absorption of cholesterol and plant sterols from the gut and decreased bile clearance of the sterols and their metabolites. Her LFTs and lipid profile returned to baseline six months after stopping *β*-sitosterol supplements and starting ezetimibe (Table 2).

Patient's LFTs remained stable for about 4 years before she was started on azathioprine for worsening liver function. A year later, she underwent liver transplantation and remained stable thereafter.

### 2.2. Case  2

A 41-year-old Caucasian female with past medical history of Turner's syndrome, hypothyroidism, type 2 diabetes mellitus, and asthma, initially found to have abnormal LFTs when she was 28 years old, presents for regular follow-up. Physical examination showed a short statured female without jaundice. Abdomen was soft and liver was palpable 2 cm below the costal margin. Laboratory data is shown in Table 1. Other lab tests including AMA, ASMA, alpha-1 antitrypsin, rapid plasma regain, serum ferritin, and serum ceruloplasmin were unremarkable. A percutaneous liver biopsy showed multiple noncaseating granulomas within portal triad and hepatic lobules; acid-fast bacilli, fungal, and iron stains were negative. LFTs were followed periodically and ALP remained high in 300–400 U/L range. A few years later, patient developed intense pruritus with worsening of her LFTs. Laboratory data showed ALT 159 U/L, AST 88 U/L, total bilirubin 0.5 mg/dL, and ALP 614 U/L. Possible hepatotoxic medications including enalapril and glipizide were discontinued without any improvement in the symptoms or LFTs. A repeat liver biopsy showed intact liver architecture without significant steatosis and multiple noncaseating granulomas; and acid-fast bacilli, fungal, and iron stains were negative. Patient had worsening pruritus that was resistant to ursodiol, cholestyramine, and hydroxyzine. To prevent worsening hyperglycemia with underlying diabetes mellitus, steroids were avoided and the patient was started on an immunosuppressant, azathioprine. With the titration of azathioprine dose up to 75 mg/day, pruritus subsided and LFTs returned to normal except ALP that remained slightly over 200 U/L.

### 2.3. Case  3

A 56-year-old African American female with past medical history of Turner's syndrome, cardiac valvular abnormalities, hypertension, type 2 diabetes mellitus, glucose-6-phosphate dehydrogenase deficiency, celiac sprue, and chronic pancreatitis with pancreatic insufficiency had biopsy-proven granulomatous hepatitis when she was 32 years old. She had abnormal LFTs, mild hepatomegaly, and splenomegaly but remained asymptomatic for several years before she presented with right upper quadrant abdominal pain, lower extremity edema, and encephalopathy. Physical exam revealed soft abdomen with right upper quadrant tenderness without rebound tenderness, guarding, or ascites. Laboratory data is shown in Table 1. Other laboratory tests included white blood cell count 4,500/*μ*L, hemoglobin 11.2 g/dL, hematocrit 35.2%, and platelet count 57,000/*μ*L. Serum AMA was negative. With worsening liver disease, she was evaluated for orthotopic liver transplant but declined surgery secondary to multiple comorbidities. Patient expired within a year of evaluation secondary to end-stage liver disease.

## 3. Discussion

Abnormal LFTs are often seen in the absence of clinical signs and symptoms of overt liver disease in patients with Turner's syndrome. One study found elevated liver enzymes in 80% of middle-aged women with Turner's syndrome but could not associate these findings with overt hepatic disease [7]. Women with Turner's syndrome do not seem to consume more alcohol than other women [8]. Although both excess weight and estrogen replacement therapy have been suggested as the cause of abnormal LFTs, there is no definitive evidence of their association in Turner's syndrome [9, 10]. Autoimmune mechanism has also been suggested in some cases [9]. In a study, liver biopsies were performed in 27 patients with Turner's syndrome for persistently elevated liver enzymes [7]. Ten out of 27 patients had marked architectural liver changes including nodular regenerative hyperplasia (*n* = 6), multifocal nodular hyperplasia (*n* = 2), and cirrhosis (*n* = 2). Other patients showed more moderate changes, including portal fibrosis, inflammatory infiltrates, and nonalcoholic fatty liver disease. The authors concluded that the main causes of liver abnormalities in Turner's syndrome were vascular disorders related to congenitally abnormal vessels and metabolic disorders leading to steatosis, steatofibrosis, and steatohepatitis. They could not find any evidence of liver toxicity from estrogen replacement therapy.

Association of Turner's syndrome with sarcoidosis and granulomatous process has been described previously [6]. In a case report, a 32-year-old Japanese woman with Turner's syndrome was found to have elevated LFTs and serum ACE levels and was found to have sarcoidosis based on the noncaseating granulomas on lymph node biopsy. An autoimmune disorder such as hypothyroidism is extremely common in Turner's syndrome [1, 6]. Of the 3 patients, two of our patients had hypothyroidism and one had celiac sprue. There are few reports of association of sarcoidosis and autoimmune diseases. Several possible mechanisms have been suggested for this association [11–14]. Based on this data, the granulomatous process could be related to the autoimmune disorders that are commonly seen in patients with Turner's syndrome. The association between Turner's syndrome and inflammatory bowel disease is also well reported. All three of our patients had endoscopies that ruled out inflammatory bowel disease.

Our first patient in the case series had a rare disorder, sitosterolemia, which manifested when she consumed alternative supplements containing plant sterols. In sitosterolemia, the genetic defect is expressed at two levels. In the intestine, there is a failure to distinguish between luminal/dietary cholesterol and noncholesterol sterols and hence both are absorbed. In the liver, there is an inability to excrete sterols, either cholesterol or noncholesterol sterols, into the bile [15]. Cholestatic liver damage has not been reported in known cases of sitosterolemia [16]. There is only one published case report with sitosterolemia presenting with progressive liver disease, initially categorized as chronic active hepatitis leading to cirrhosis, and later found to have sitosterolemia that improved dramatically after orthotopic liver transplantation [15]. In our patient, LFTs worsened upon ingestion of *β*-sitosterol supplements and improved after discontinuation of these supplements suggesting a temporal and causative association. However, she did not have a genetic testing to confirm sitosterolemia. Hence she might have had high sitosterol levels based on excess intake and underlying cholestasis.

Our case series highlight the wide spectrum of granulomatous liver disease in patients with Turner's syndrome. First patient underwent liver transplantation; second patient developed worsening LFTs with pruritus that was successfully treated with an immunosuppressant; and third patient had progressively worsening liver disease that eventually led to her death.

In conclusion, elevated liver enzymes are frequently observed in Turner's syndrome. Hepatic granulomas, which have never been described in association with Turner's syndrome previously, should be considered as a possible cause of abnormal LFTs in patients with Turner's syndrome. Although hepatic granulomas are usually clinically silent with LFTs demonstrating chronic cholestatic pattern to systemic symptoms such as pruritus, portal hypertension and cirrhosis are rare complications of hepatic granulomas [17] and it is imperative to be aware of these complications as they can be life threatening.

## Figures and Tables

**Table 1:** 

Laboratory test	Case report 1	Case report 2	Case report 3	Normal
Alanine transaminase (ALT)	223	116	103	13–69 U/L
Aspartate transaminase (AST)	113	81	125	15–46 U/L
Alkaline phosphatase (ALP)	404	330	512	38–126 U/L
Total bilirubin/direct bilirubin	1.2/0.4	0.7	4.9/4	0.2–1.3 mg/dL
Hepatitis: B, C panel	Negative	Negative	Negative	Negative
Serum angiotensin converting enzyme (ACE)	60	Normal	53	8–52 U/L
Anti-nuclear antibody	Negative	Negative	Negative	Negative

**Table 2:** 

Laboratory test	On *β*-sitosterol supplements	Six months after stopping *β*-sitosterol supplements and starting ezetimibe	Normal values
ALT	458	202	13–69 U/L
AST	211	87	15–46 U/L
ALP	528	371	38–126 U/L
Total bilirubin	5.4	1.8	0.2–1.3 mg/dL
Direct bilirubin	2.7	0.8	0–0.3 mg/dL
Total cholesterol	857	338	<150 mg/dL
Low density cholesterol (LDL)	823	255	<100 mg/dL
High density cholesterol (HDL)	17.7	59	>40 mg/dL
Triglycerides	82	121	<150 mg/dL
Albumin	3.0	3.2	3.5–5.5 mg/dL
